# Low-Pressure Hydrocephalus in Spontaneous Angiogram-Negative Subarachnoid Hemorrhage Following COVID-19 Infection

**DOI:** 10.7759/cureus.16674

**Published:** 2021-07-27

**Authors:** Luke Weisbrod, Caroline Davidson, Andrew Gard, Daniel Surdell

**Affiliations:** 1 Neurosurgery, University of Nebraska Medical Center, Omaha, USA

**Keywords:** covid-19, iatrogenic subarachnoid hemorrhage, low-pressure hydrocephalus, intraventricular hematoma, sars cov-2 infection

## Abstract

A preliminary report warned that severe acute respiratory syndrome coronavirus 2 (SARS-CoV-2) could have neuro-invasive potential as it was observed that some patients showed neurologic symptoms such as headache, nausea, and vomiting. Following early speculation there have been reports of neurologic manifestations involving both the central nervous system and peripheral nervous system including reports that coronavirus disease 2019 (COVID-19) may increase the risk of acute ischemic stroke. Here we present a patient with recent COVID-19 infection who experienced low-pressure hydrocephalus requiring high-output cerebrospinal fluid (CSF) diversion following spontaneous angiogram-negative subarachnoid hemorrhage. We hypothesize that patients who are either currently or who have recently been infected with SARS-CoV-2 may have altered ventricular compliance and/or altered CSF hydrodynamics from mechanisms that are not yet understood but potentially related to previously described pathophysiologic mechanisms of the virus and associated inflammatory reaction.

## Introduction

Coronavirus disease 2019 (COVID-19) is caused by severe acute respiratory disease syndrome coronavirus 2 (SARS-CoV-2) infection and has been declared a Public Health Emergency of International Concern by the World Health Organization [1]. A preliminary report warned that SARS-CoV-2 could have neuro-invasive potential as it was observed that some patients showed neurologic symptoms such as headache, nausea, and vomiting [2]. Following early speculation there have been reports of neurologic manifestations involving both the central nervous system (CNS) and peripheral nervous system (PNS) [3] including reports that COVID-19 may increase the risk of acute ischemic stroke [4]. Here we present the case of a patient with recent COVID-19 infection who experienced low-pressure hydrocephalus requiring high-output cerebrospinal fluid (CSF) diversion following spontaneous angiogram-negative subarachnoid hemorrhage (SAH).

## Case presentation

**Figure 1:**
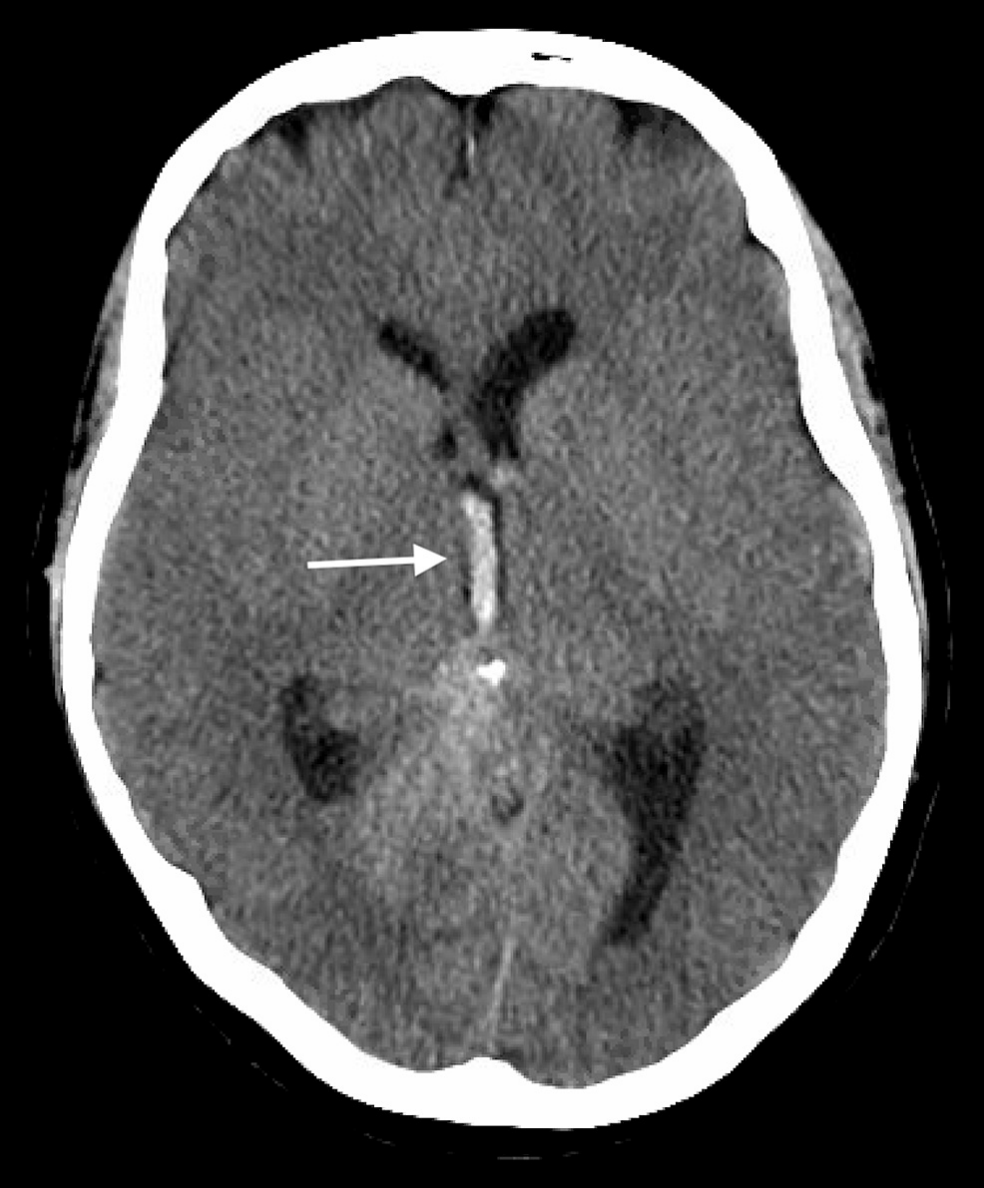
Axial non-contrasted CT head with evidence of intra-ventricular hemorrhage within the third ventricle and early ventriculomegaly (arrow). CT, computed tomography.

**Figure 2:**
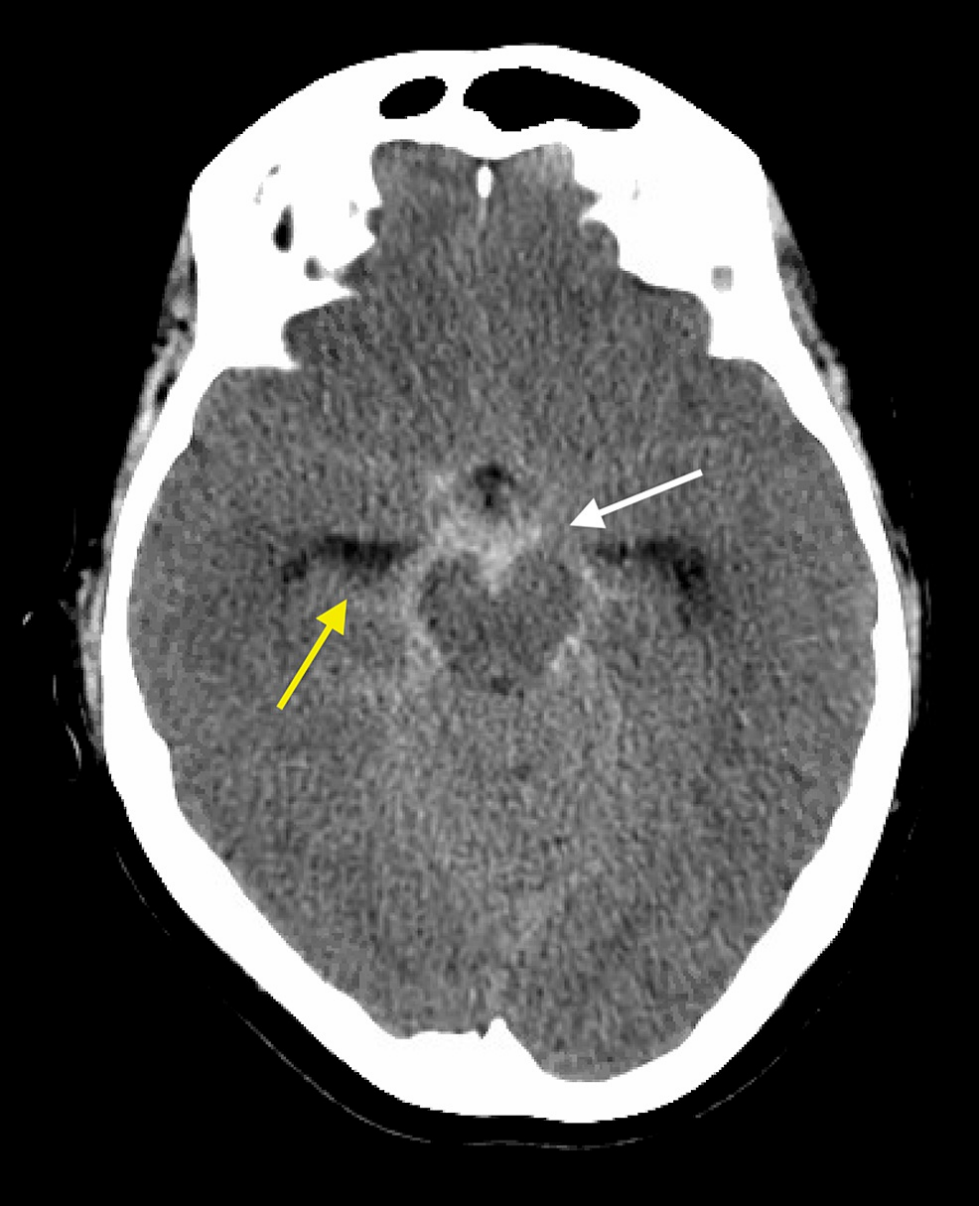
Axial non-contrasted CT head demonstrating diffuse subarachnoid hemorrhage throughout the basilar cisterns (white arrow) and enlargement of the ventricular temporal horns (yellow arrow). CT, computed tomography.

**Figure 3:**
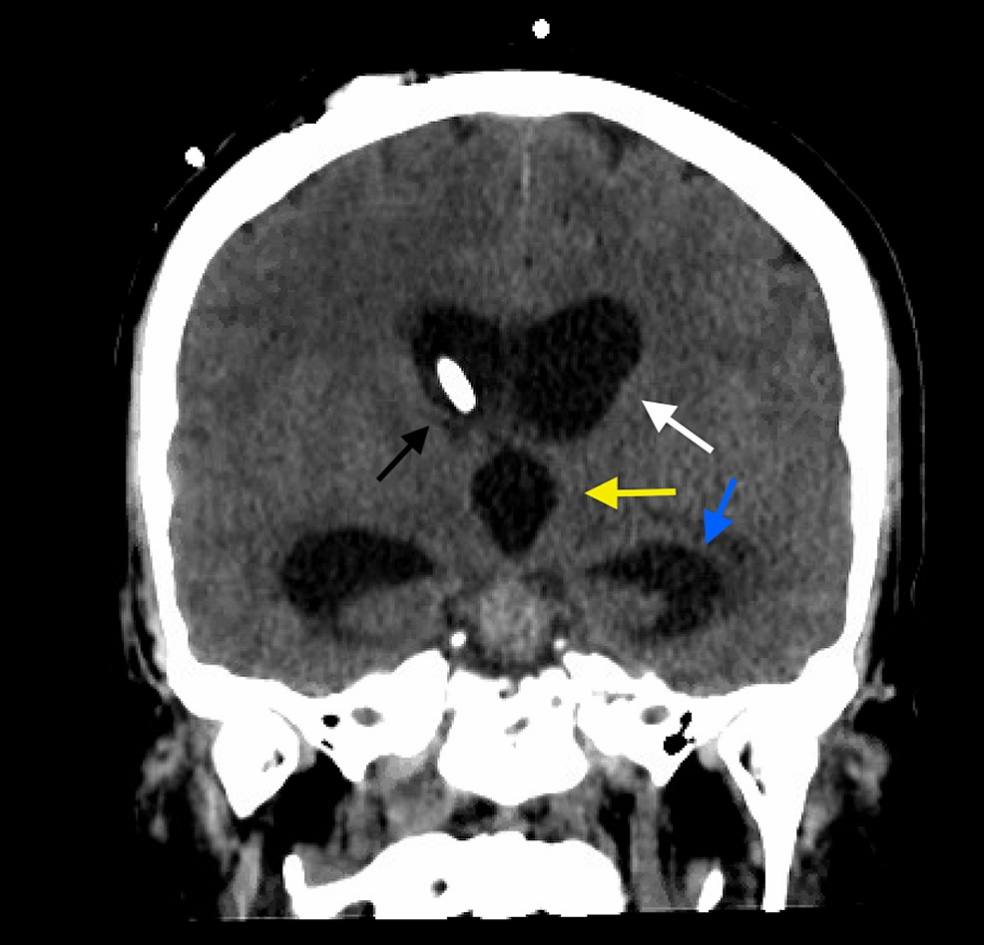
Coronal non-contrasted CT head demonstrating ventriculomegaly of the third lateral ventricle (white arrow), third ventricle (yellow arrow), and temporal horns (blue arrow) despite functioning EVD terminating in right foramen of Monro (black arrow). CT, computed tomography; EVD, external ventricular drain.

**Figure 4:**
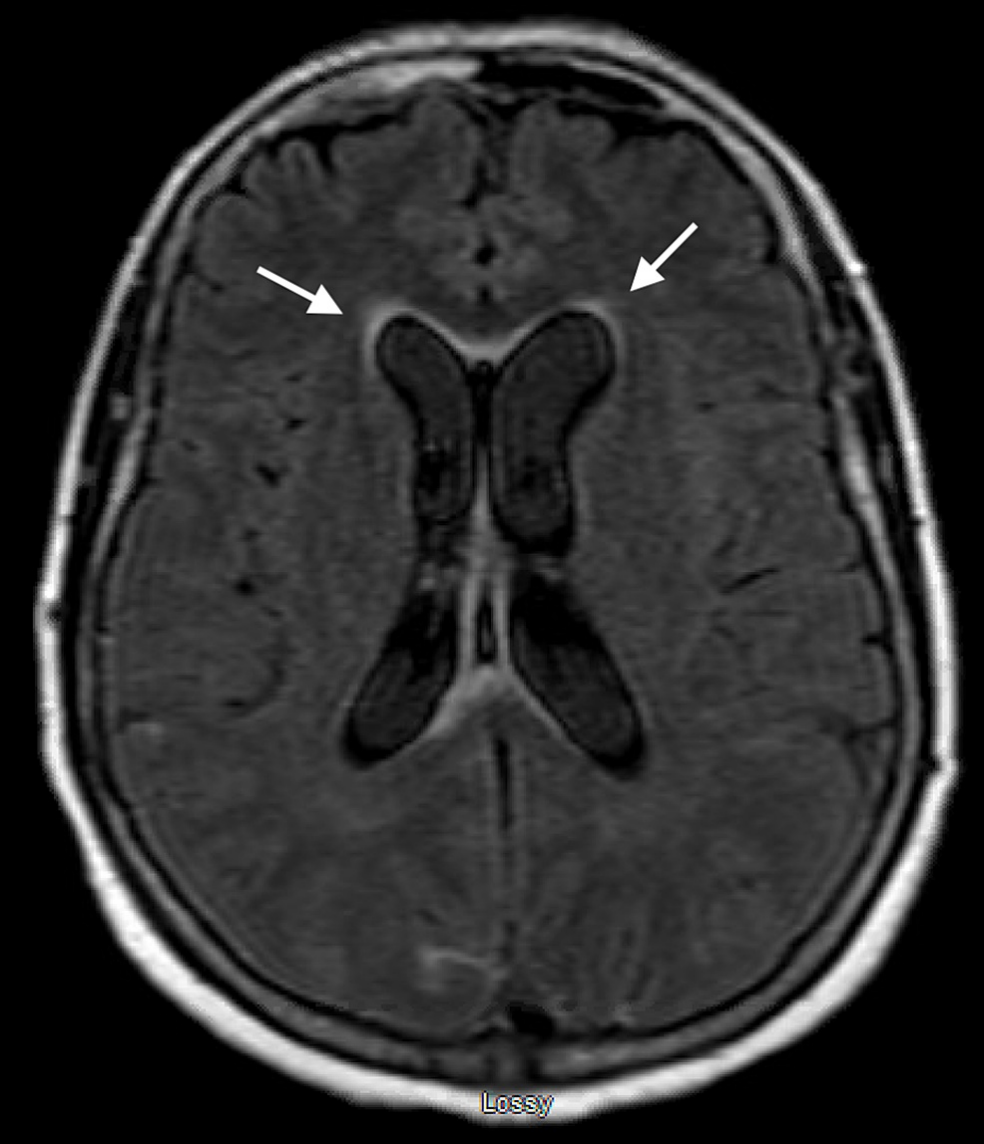
MRI brain FLAIR sequence demonstrating ventriculomegaly in the bilateral lateral ventricles with associated transependymal edema. MRI, magnetic resonance imaging; FLAIR, fluid-attenuated inversion recovery.

**Figure 5:**
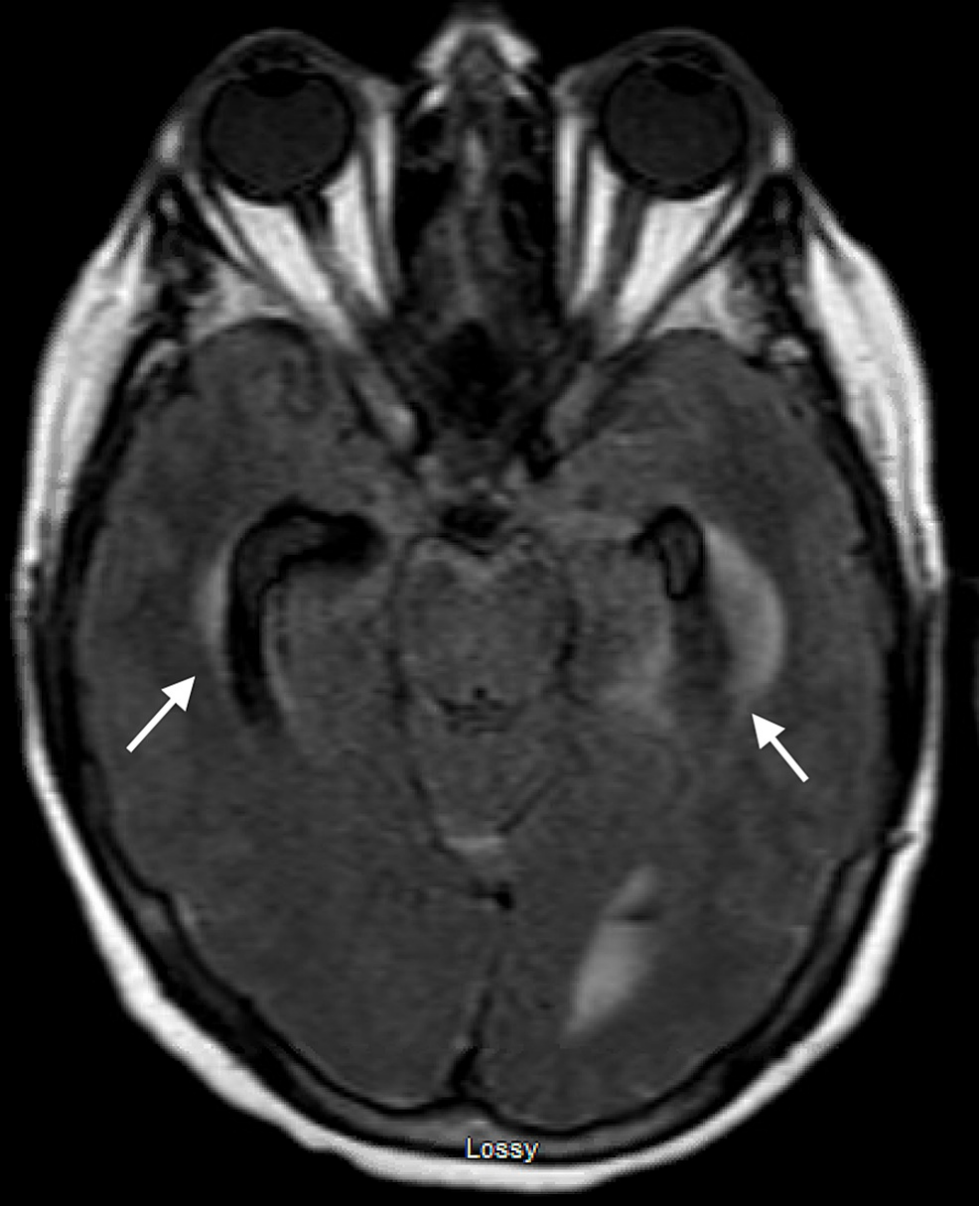
MRI brain FLAIR sequence demonstrating enlargement of the bilateral temporal horns of the ventricles with associated transependymal edema. MRI, magnetic resonance imaging; FLAIR, fluid-attenuated inversion recovery.

We present the case of a 62-year-old female with a significant past medical history of well-controlled hypertension on monotherapy with metoprolol and a recent symptomatic COVID-19 infection one month prior to admission in early November of 2020 diagnosed by polymerase chain reaction. Her COVID-19 symptoms progressed over the course of around seven to 10 days from generalized malaise to headaches and ultimately intermittent fevers. She did not experience upper respiratory symptoms such as cough or shortness of breath, nor did she experience anosmia. She did not require inpatient admission or specific treatment for her COVID-19 infection. Following recovery from her infection she presented as a transfer to our facility hours after sudden onset of severe headache, nausea, and vomiting while leaning over doing light housework. She decided to seek medical attention for this headache due to the headache severity, stating that it was "the worst headache of my life." On arrival to the outside facility her blood pressure was elevated into the 170s systolic requiring treatment with additional anti-hypertensive agents. Radiographically she was found to have diffuse SAH throughout the basilar cisterns, intraventricular hemorrhage, and evidence of hydrocephalus on computed tomography (CT) head without contrast (Figures 1, 2). She had no focal neurologic deficits on admission and clinically her grade was categorized as World Federation of Neurologic Surgeon grade 1 and Hunt and Hess grade 2. Radiographically she was classified as Modified Fisher grade 2. An external ventricular drain (EVD) was placed shortly after admission for CSF diversion and was leveled to 15 cm of water (H_2_O). Following placement of the EVD she underwent further diagnostic evaluation with a computed tomography angiography (CTA) of the head and neck, which was negative for aneurysm, vascular malformation, dissection, or other identifiable etiology for her SAH. Digital subtraction angiography on post-bleed day 1 and magnetic resonance imaging (MRI) on post-bleed day 2 were also negative for identifiable underlying etiology for the SAH. Her mental status progressively declined during the days following admission to the point that she would not open her eyes unless stimulated by noxious stimuli and was completed disoriented, which was noted to be in concordance with progressive increase in ventricular size and trans-ependymal edema (Figure 3) despite CSF diversion by way of a functioning EVD. Her MRI brain demonstrates these findings as well (Figures 4, 5). 

**Figure 6:**
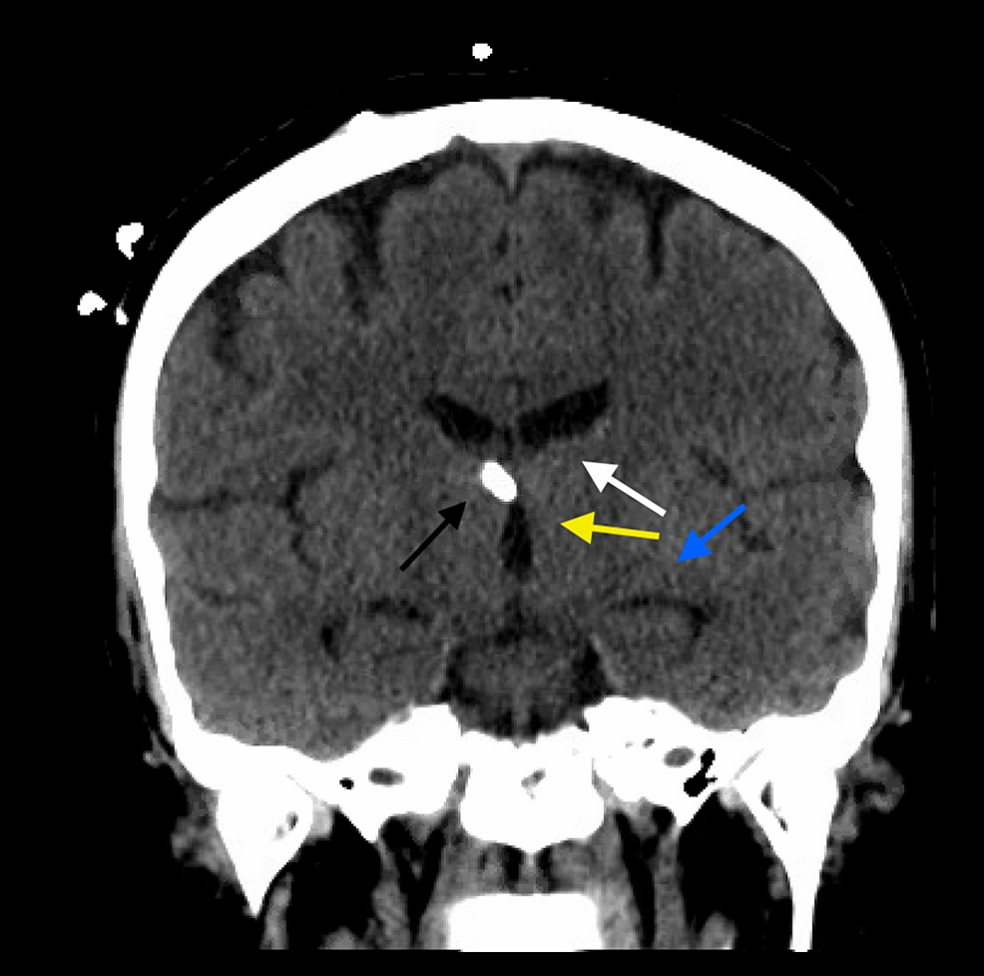
Coronal non-contrasted CT head demonstrating interval decrease in the size of lateral ventricle (white arrow), third ventricle (yellow arrow), and temporal horns (blue arrow) following functioning EVD lowered to 0 cm H2O terminating in right foramen of Monro (black arrow). CT, computed tomography; EVD, external ventricular drain.

Only once her EVD was progressively lowered to 0 cm H_2_O did her mental status improve and ventricular size begin to decrease on follow-up CT imaging. Her EVD was ultimately successfully weaned and removed on post-bleed day 15 without the need for permanent CSF diversion in the form of a ventriculoperitoneal shunt (Figure 6). Shortly after EVD removal she was discharged home with home health care. She underwent a repeat CTA head and neck prior to discharge, which remained negative for identifiable etiology for her SAH. Her three-month follow-up CTA head and neck remained negative for identifiable aneurysm and otherwise demonstrated complete resolution of intracranial hemorrhage and no evidence of delayed hydrocephalus. 

## Discussion

SAH is a common problem encountered by neurosurgeons. The estimated annual incidence of spontaneous SAH in the United States is 9.7-14.5 per 100,000 [5,6]. Spontaneous, non-traumatic SAH is most commonly caused by saccular aneurysms with arteriovenous malformations, arterial dissections, tumors, and other vascular abnormalities such as vasculitis causing SAH in the minority of cases [7]. Occasionally, no structural etiology for the SAH can be identified on radiographic imaging. These hemorrhages, termed idiopathic SAH or angiogram-negative SAH, represent approximately 15% of spontaneous SAH [8-13]. The incidence of patients requiring CSF diversion in angiogram-negative SAH is around 15.6% with 5.6% of patients requiring permanent CSF diversion in the form of a shunt [14]. We present a case of angio-negative SAH with significantly altered CSF mechanics following a recent SARS-CoV-2 infection. 

The binding of SARS-CoV-2 to angiotensin-converting enzyme (ACE2) is a critical step in the pathophysiology of clinical manifestations in patients with COVID-19 [15]. The function of ACE2 in normal human physiology is to regulate blood pressure via inhibition of the angiotensin-renin-aldosterone pathways [15]. ACE2 has a wide distribution in multiple organs including the nose, lungs, kidneys, liver, blood vessels, immune system, and the brain [16]. After binding to ACE2, downstream effects can include damage to mitochondria and lysosomes resulting in protein misfolding, protein aggregation, and cell death [17]. By SARS-CoV-2 binding to ACE2, it can inhibit the metabolic conversion of angiotensin-2 to angiotensin-1, resulting in higher levels of pro-inflammatory markers, vasoconstriction, vascular permeability, edema, and vascular injury [17].

Following early speculation there have been reports of neurologic manifestations involving both the CNS and PNS [3]. Cytokine activation in the CNS can result in small and large vessel occlusions, leading to acute ischemic stroke or cerebral venous sinus thrombosis if in an artery or vein/sinus, respectively [17]. Additionally, cytokine activation can result in damage to the blood-brain barrier and injure neuroglia once infiltrated into the CNS either directly or as a result of formation of autoantibodies by a mechanism known as molecular mimicry [17]. We hypothesize that patients who are either currently or who have recently been infected with SARS-CoV-2 may have altered ventricular compliance and/or altered CSF hydrodynamics from mechanisms that are not yet understood but are potentially related to one or multiple of the aforementioned mechanisms. 

There have been reports of both spontaneous non-aneurysmal as well as spontaneous aneurysmal SAH in patients acutely infected with COVID-19 [18,19]. To our knowledge, this is the first reported case of spontaneous non-aneurysmal SAH with associated low-pressure hydrocephalus in the setting of recent COVID-19 infection. It is possible that the correlation is merely coincidental; however, it is difficult to know with certainty as the neurologic manifestations related to COVID-19 continue to grow in the literature. We, therefore, saw it to be prudent to report our findings. We did not send CSF in our case as our facility’s laboratory was not equipped to run COVID-19 testing on CSF samples. It has been suggested, however, that the current testing of COVID-19 in CSF may not be an accurate marker for neurologic manifestations in the setting of COVID-19 as only around 6% of patients with acute COVID-19 infection and neurologic manifestations had CSF that tested positive for COVID-19 [20]. This potentially suggests that more accurate CSF biomarkers need to be developed to detect COVID-19 or the neurologic manifestations of COVID-19 act in a route that is not directly involved in the CSF but may still have implications on CSF hydrodynamics. 

For similar patients with non-aneurysmal SAH with low-pressure hydrocephalus, we would recommend follow-up in three months with a repeat CTA head/neck in search of etiology for SAH as well as to follow up on hydrocephalus. Consideration could also be given to involving infectious disease and potentially obtaining a CSF sample if testing is immediately available at the provider's institution. 

## Conclusions

In this study, we report a case of post-COVID-19 infection angio-negative SAH with significantly altered CSF hydrodynamics resulting in low-pressure hydrocephalus. There have been multiple reports of COVID-19 resulting in CNS manifestations. The mechanism and inflammatory reaction resulting from SARS-CoV-2 resulting in other neurologic manifestations may also contribute to alterations in CSF hydrodynamics leading to a new set of therapeutic challenges in SAH management.

## References

[1] (2020). World Health Organization. Statement on the Second Meeting of the International Health Regulations (2005) Emergency Committee Regarding the Outbreak of Novel Coronavirus (2019-nCoV). https://www.who.int/news-room/detail/30-01-2020-statement-on-the-second-meeting-of-the-international-health-regulations-(2005)-emergency-committee-regarding-the-outbreak-of-novel-coronavirus-(2019-ncov).

[2] Li YC, Bai WZ, Hashikawa T (2020). The neuroinvasive potential of SARS-CoV2 may play a role in the respiratory failure of COVID-19 patients. J Med Virol.

[3] Tsivgoulis G, Palaiodimou L, Katsanos AH (2020). Neurological manifestations and implications of COVID-19 pandemic. Ther Adv Neurol Disord.

[4] Hassett C, Gedansky A, Mays M, Uchino K (2020). Acute ischemic stroke and COVID-19. Cleve Clin J Med.

[5] Burns JD, Huston J 3rd, Layton KF, Piepgras DG, Brown RD Jr (2009). Intracranial aneurysm enlargement on serial magnetic resonance angiography: frequency and risk factors. Stroke.

[6] Miyazawa N, Akiyama I, Yamagata Z (2006). Risk factors for growth of unruptured intracranial aneurysms: follow-up study by serial 0.5-T magnetic resonance angiography. Neurosurgery.

[7] Al-Mufti F, Merkler AE, Boehme AK (2018). Functional outcomes and delayed cerebral ischemia following nonperimesencephalic angiogram-negative subarachnoid hemorrhage similar to aneurysmal subarachnoid hemorrhage. Neurosurgery.

[8] Sarabia R, Lagares A, Fernandez-Alen JA (2010). Idiopathic subarachnoid hemorrhage: a multicentre series of 220 patients. Neurocirugia (Astur).

[9] Hui FK, Schuette AJ, Moskowitz SI, Gupta R, Spiotta AM, Obuchowski NA, Cawley CM (2011). Antithrombotic states and outcomes in patients with angiographically negative subarachnoid hemorrhage. Neurosurgery.

[10] Alexander MS, Dias PS, Uttley D (1986). Spontaneous subarachnoid hemorrhage and negative cerebral panangiography. Review of 140 cases. J Neurosurg.

[11] van Gijn J, van Dongen KJ, Vermeulen M, Hijdra A (1985). Perimesencephalic hemorrhage: a nonaneurysmal and benign form of subarachnoid hemorrhage. Neurology.

[12] Van Calenbergh F, Plets C, Goffin J, Velghe L (1993). Nonaneurysmal subarachnoid hemorrhage: prevalence of perimesencephalic hemorrhage in a consecutive series. Surg Neurol.

[13] Whiting J, Reavey-Cantwell J, Velat G, Fautheree G, Firment C, Lewis S, Hoh B (2009). Clinical course of nontraumatic, nonaneurysmal subarachnoid hemorrhage: a single-institution experience. Neurosurg Focus.

[14] Boswell S, Thorell W, Gogela S, Lyden E, Surdell D (2013). Angiogram-negative subarachnoid hemorrhage: outcomes data and review of the literature. J Stroke Cerebrovasc Dis.

[15] Verdecchia P, Cavallini C, Spanevello A, Angeli F (2020). The pivotal link between ACE2 deficiency and SARS-CoV-2 infection. Eur J Intern Med.

[16] Li MY, Li L, Zhang Y, Wang XS (2020). Expression of the SARS-CoV-2 cell receptor gene ACE2 in a wide variety of human tissues. Infect Dis Poverty.

[17] Fotuhi M, Mian A, Meysami S, Raji CA (2020). Neurobiology of COVID-19. J Alzheimers Dis.

[18] Harrogate S, Mortimer A, Burrows L, Fiddes B, Thomas I, Rice CM (2021). Non-aneurysmal subarachnoid haemorrhage in COVID-19. Neuroradiology.

[19] Dodd WS, Jabbour PM, Sweid A (2021). Aneurysmal subarachnoid hemorrhage in COVID-19 patients: a case series. World Neurosurg.

[20] Lewis A, Frontera J, Placantonakis DG, Lighter J, Galetta S, Balcer L, Melmed KR (2021). Cerebrospinal fluid in COVID-19: a systematic review of the literature. J Neurol Sci.

